# Editorial: Ethnomedicinal strategies for controlling pathogen colonization in livestock: integrating traditional practices into modern livestock health management

**DOI:** 10.3389/fvets.2026.1793301

**Published:** 2026-02-10

**Authors:** Deji A. Ekunseitan, Ibukun M. Famuyide, Oludotun O. Adelusi, Akeem A. Ayoola

**Affiliations:** 1 Department of Animal Sciences, North Carolina Agricultural and Technical State University, Greensboro, NC, United States; 2 Council for Scientific and Industrial Research (CSIR), Pretoria, South Africa; 3 Department of Animal Production and Health, Federal University of Agriculture, Abeokuta, Ogun State, Nigeria

**Keywords:** animal health, ethnomedicinal plants, foodborne pathogens, gut microbiome, phytogenics

## Introduction

The intensification of livestock production over recent decades has been accompanied by remarkable gains in productivity, efficiency, and global food security. However, these advances have also intensified long-standing challenges related to infectious diseases, foodborne pathogens, and the extensive reliance on antimicrobial agents. For decades, antibiotics have served as indispensable tools for disease control and productivity enhancement in poultry, dairy, and other livestock systems. However, the widespread therapeutic and prophylactic use of antimicrobials has accelerated the emergence of multidrug-resistant pathogens, compromised treatment efficacy, and increased the threshold of antimicrobial residues entering the food chain. These challenges have catalyzed a renewed search for sustainable, non-antibiotic strategies that can maintain animal health while aligning with One Health objectives.

The global livestock sector is undergoing a critical transition driven by intensifying concerns over antimicrobial resistance (AMR), food safety, animal welfare, and environmental sustainability. Antimicrobial resistance (AMR), now recognized as a global One Health crisis, threatens not only animal health and productivity but also human health and environmental sustainability. Within this context, the Research Topic provides a timely and scientifically grounded platform for re-evaluating traditional veterinary knowledge through the lens of modern experimental science. The articles assembled in this topic collectively demonstrate that ethnomedicinal strategies are no longer confined to anecdotal or historical relevance. Instead, they are increasingly supported by robust *in vivo* experimentation, molecular immunology, microbiome profiling, and systems-level analyses. Together, these contributions advance the field beyond the simple replacement of antibiotics, positioning ethnomedicine as a complementary and integrative approach capable of reshaping livestock health management in an era defined by AMR, consumer pressure for residue-free products, and the need for sustainable production systems. Rather than positioning ethnomedicine as an alternative rooted solely in tradition, the articles in this Research Topic emphasize integration, mechanistic understanding, and translational relevance across livestock production systems.

## Ethnomedicine as a strategic response to antimicrobial resistance

AMR represents one of the most pressing threats to animal and public health worldwide. In poultry production, pathogens such as *Salmonella enterica* serovars continue to exhibit resistance to commonly used antibiotics, undermining both disease control and food safety. Similarly, in dairy systems, mastitis-causing pathogens, including methicillin-resistant *Staphylococcus aureus* and multidrug-resistant streptococci, have reduced the effectiveness of conventional therapies while increasing the risk of antibiotic residues in milk. A unifying theme across the articles is the urgent need to reduce dependence on traditional antibiotics without compromising animal welfare or production efficiency. In poultry and dairy systems alike, the irrational or prophylactic use of antimicrobials has driven the emergence of multidrug-resistant pathogens such as *Salmonella* spp., *Staphylococcus aureus, Streptococcus uberis*, and *Streptococcus dysgalactiae*. The studies highlighted in this Research Topic directly address this challenge by evaluating ethnomedicinal interventions that exert antimicrobial, anti-inflammatory, and immunomodulatory effects through multiple, often synergistic mechanisms. Unlike the traditional antibiotics, which typically act on single pathogen targets, plant-derived bioactive compounds and traditional formulations often exhibit broad spectrum of action that influence a network of biological pathways. This includes modulation of host immune responses, reinforcement of epithelial barrier integrity, alteration of gut or mammary microbiota composition, and suppression of pathogen virulence factors. Such multi-target effects reduce the likelihood of resistance development while simultaneously improving host resilience to infection.

## Controlling pathogen colonization and enhancing gut integrity

Pathogen colonization, rather than clinical disease alone, represents a critical control point for improving animal health and food safety. Subclinical intestinal colonization by *Salmonella* in poultry or persistent intramammary infections in dairy cattle often serve as reservoirs for environmental contamination and zoonotic transmission. Zhang et al. emphasized the importance of ethnomedicinal strategies particularly in their effectiveness in host-microbe interface. Experimental evidence demonstrates that ethnoveterinary plant supplements such as *Rauwolfia serpentina* root powder can reduce intestinal pathogen loads, improve gut morphology, and regulate immune-related gene expression in poultry challenged with *Salmonella Gallinarum*. These effects are accompanied by improved growth performance and feed efficiency, highlighting the dual benefits of disease control and production enhancement. Importantly, these findings challenge the misconception that natural products are inherently less effective than antibiotics, demonstrating that properly formulated and dosed ethnomedicinal interventions can deliver measurable, biologically and meaningful outcomes. The mixture of three medicinal plants *Sarcococca ruscifolia, Hedera nepalensis var. sinensis*, and *Clematis chinensis* by Yang et al. (1) as a dietary supplement improved intestinal health and antioxidant capacity. The plant powder reduced expression of pro-inflammatory cytokine TNF-α, suggesting an immunomodulatory benefit. These findings are important as they highlight a natural, low-toxicity strategy for supporting poultry gut integrity an important aspect of resilience against pathogens and overall flock productivity.

## Ethnomedicine and mastitis control in dairy cattle

Complementing the poultry-focused research, a comprehensive review by Fan et al. examined traditional Chinese medicine (TCM) and plant-derived bioactive compounds as sustainable alternatives to antibiotics in bovine mastitis management. Mastitis remains one of the most economically devastating diseases in the dairy industry, accounting for substantial losses due to reduced milk yield, compromised milk quality, and increased veterinary costs. The review synthesizes evidence showing that numerous medicinal plants and TCM formulations exert antibacterial and anti-inflammatory effects against mastitis pathogens while modulating host immune responses. By targeting inflammatory signaling pathways such as NF-κB, MAPK, and PI3K-AKT, these interventions reduce tissue inflammation, limit bacterial persistence, and improve milk quality while minimizing the risk of antibiotic residues and improving clinical outcomes. The emerging concept of the “intestinal-mammary axis” further expands the relevance of ethnomedicine, suggesting that modulation of gut microbiota may indirectly influence mammary health and disease susceptibility. Notably, such approaches may also mitigate concerns associated with antibiotic residues in milk, a critical public health issue. Ethnomedicinal strategies capable of reshaping the intestinal microbiome may therefore exert indirect protective effects against mastitis, further expanding their relevance within integrated livestock health management frameworks.

## Mechanistic validation and the rise of omics-enabled ethnomedicine

One of the most significant strengths of this Research Topic is its emphasis on mechanistic validation. The integration of transcriptomics, metabolomics, and microbiome analyses represents a critical step toward transforming ethnomedicinal practices into standardized, evidence-based tools suitable for modern livestock systems. Providing a systems level insight into how ethnomedicinal bioactive compounds influence host physiology and pathogen dynamics. For example, alterations in inflammatory cytokine profiles, oxidative stress markers, and metabolic pathways have been linked to improved disease resistance and performance outcomes. Zhao et al. demonstrated how plant-derived compounds influence immune-related gene expression, oxidative stress pathways, and metabolic networks linked to inflammation and pathogen resistance. This mechanistic approach not only strengthened scientific credibility but also facilitates regulatory acceptance and rational formulation of ethnomedicinal products. By identifying specific bioactive compounds, signaling pathways, and host responses, these studies move the field away from empirical usage toward precision ethnomedicine. Such advances open the door to synergistic applications that combine traditional knowledge with advanced technologies, including nano-delivery systems, artificial intelligence driven formulation optimization, and predictive modeling of host microbiome interactions.

## Socioeconomic, environmental, and one health implications

Beyond biological efficacy, these articles highlight the broader socioeconomic and environmental relevance of ethnomedicinal strategies. Many medicinal plants evaluated in these studies are locally available, culturally accepted, and environmentally sustainable, particularly in low and middle income countries where access to conventional therapeutics may be limited. Their use can reduce dependence on traditional antimicrobials, lower production costs, and enhance resilience in smallholder and commercial livestock systems alike. From a One Health perspective, ethnomedicinal approaches contribute to reduced antimicrobial residues in animal products, lower environmental contamination with antibiotic-resistant bacteria, and diminished risk of zoonotic transmission. These benefits align closely with global policy initiatives aimed at curbing AMR and promoting sustainable agri-food systems. Importantly, it underscores that successful implementation will require collaboration among researchers, veterinarians, farmers, policymakers, and regulatory agencies to ensure quality control, farmer adoption, and harmonized standards.

## Challenges and future directions

Despite the promising findings presented, there exist some key challenges that must be addressed to fully realize the potential of these strategies. Variability in plant composition, differences in extraction methods, and inconsistencies in dosing remain significant obstacles. Large-scale, multi-site studies are needed to validate efficacy under diverse management conditions and genetic backgrounds. Long-term safety assessments and evaluation of potential interactions with traditional therapeutics are equally essential. Future research should prioritize integrative frameworks that combine ethnomedicine with nutrition, management, vaccination, and selective antimicrobial use. Such holistic approaches are more likely to deliver durable health outcomes than single interventions applied in isolation. The incorporation of omics technologies and systems biology will be instrumental in identifying biomarkers of response and tailoring interventions to specific production systems.

## Conclusion

The articles featured in “*Ethnomedicinal Strategies for Controlling Pathogen Colonization in Livestock”* collectively provide compelling evidence that traditional veterinary knowledge, when rigorously evaluated and thoughtfully integrated, can play a transformative role in modern livestock health management. By addressing pathogen colonization, AMR, immune modulation, and host microbiome interactions, ethnomedicinal strategies offer a scientifically credible, socially acceptable, and environmentally sustainable pathway toward reducing antimicrobial dependence. This Research Topic not only advances scientific understanding but also provides a blueprint model for integrating culturally rooted practices into evidence-based, offering innovative solutions to some of the most pressing challenges facing global animal agriculture. As the livestock sector navigates the complexities of AMR, food safety, and sustainability, ethnomedicine stands poised to transition from the margins to the mainstream of evidence-based animal health care.
